# Cryo-EM analysis of the post-fusion structure of the SARS-CoV spike glycoprotein

**DOI:** 10.1038/s41467-020-17371-6

**Published:** 2020-07-17

**Authors:** Xiaoyi Fan, Duanfang Cao, Lingfei Kong, Xinzheng Zhang

**Affiliations:** 1 National Laboratory of Biomacromolecules, CAS Center for Excellence in Biomacromolecules, Institute of Biophysics, Chinese Academy of Sciences, 100101 Beijing, China; 2 University of Chinese Academy of Sciences, 100049 Beijing, China; 3 Center for Biological Imaging, CAS Center for Excellence in Biomacromolecules, Institute of Biophysics, Chinese Academy of Sciences, 100101 Beijing, China

**Keywords:** SARS virus, Cryoelectron microscopy

## Abstract

Global emergencies caused by the severe acute respiratory syndrome coronavirus (SARS-CoV), Middle-East respiratory syndrome coronavirus (MERS-CoV) and SARS-CoV-2 significantly endanger human health. The spike (S) glycoprotein is the key antigen and its conserved S2 subunit contributes to viral entry by mediating host-viral membrane fusion. However, structural information of the post-fusion S2 from these highly pathogenic human-infecting coronaviruses is still lacking. We used single-particle cryo-electron microscopy to show that the post-fusion SARS-CoV S2 forms a further rotated HR1-HR2 six-helix bundle and a tightly bound linker region upstream of the HR2 motif. The structures of pre- and post-fusion SARS-CoV S glycoprotein dramatically differ, resembling that of the Mouse hepatitis virus (MHV) and other class I viral fusion proteins. This structure suggests potential targets for the development of vaccines and therapies against a wide range of SARS-like coronaviruses.

## Abstract (web summary)

The spike (S) protein of coronaviruses is responsible for receptor recognition and the fusion between the viral membrane and the of cell host membrane. Here the authors report a cryo-EM structure of SARS-CoV post-fusion S2 trimer, providing insights into the fusion mechanism that could be useful for therapeutic development against coronaviruses.

## Introduction

Coronaviruses are a family of enveloped, positive-sense, single-stranded RNA viruses that typically cause mild respiratory tract infections of humans^1^. However, the outbreaks of infections caused by severe acute respiratory syndrome coronavirus (SARS-CoV) and Middle-East respiratory syndrome coronavirus (MERS-CoV) during the past two decades caused serious epidemics and brought these highly infectious human pathogens to the forefront of research on globally significant infectious diseases^2–5^. The outbreak of coronavirus disease that begun in late 2019 (COVID-19) is caused by a newly discovered novel coronavirus (severe acute respiratory syndrome coronavirus 2, SARS-CoV-2) that at the time this paper was submitted, led to ~84,000 infections and 4600 deaths in China^6–8^. The ensuing COVID-19 global pandemic had caused more than 3 million infections by April 28, 2020. SARS-CoV, MERS-CoV, and SARS-CoV-2 are zoonotic coronaviruses that possibly originated from bats and were transmitted to humans through different intermediate hosts^9–14^. Considering the possibility of continuous contact between humans and intermediate hosts and the high mutation rate of RNA viruses, additional emergencies caused by these and potentially other undiscovered coronaviruses represent serious future threats to global public health^15^.

The key determinant for efficient animal-to-human and human-to-human transmissions of coronaviruses is the spike (S) glycoprotein located on the virus surface, which mediates virus entry into its host cell^2,16^. The coronavirus S glycoprotein is a class I viral fusion protein that exists as a homotrimer with multiple glycosylation sites. The S glycoprotein comprises an N-terminal S1 subunit that mediates host-cell receptor recognition and a C-terminal S2 subunit that is required for fusion of the membranes of the virus and its host cell^2^. After cleavage at the boundary of the S1 and S2 subunits, the S glycoprotein remains a trimer through non-covalent binding. Interaction with host receptors and proteolytic processing of the second (S2′) cleavage site induce the dissociation of S1 subunits from the spike^17,18^. The remaining S2 trimer, which is conserved among coronaviruses, undergoes a series of conformational changes to trigger membrane fusion between the membrane of the host cell and the viral envelope^16^. Because of the critical role of S glycoprotein in viral transmission, S glycoprotein is the major target of neutralizing antibodies and the key target for drug development^2,19^. Antibodies and small molecules against both the S1 and S2 subunits have been reported to inhibit viral entry into the host cell^19–25^. The high percentage of amino acid sequence identities of S2 subunits among coronaviruses suggests that S2-specific therapies may be more effective for treating a broader range of coronavirus infections^23,26^.

We and others previously determined the pre-fusion structures of the S glycoproteins of SARS-CoV and MERS-CoV, and revealed the dynamic receptor-binding domain (RBD) with a standing conformation responsible for receptor binding^20,27–29^. Similar observations have been made for SARS-CoV-2 (ref. ^30,31^). The structures of the SARS-CoV S glycoprotein complexed with the receptor angiotensin-converting enzyme 2 (ACE2) further demonstrate the interaction between the standing RBD and the receptor, which may facilitate the release of the S1 subunits^32,33^. Relatively limited structural information is available for the S2 subunit from SARS-CoV or MERS-CoV in the hemi-fusion or post-fusion state, except for the heptad repeat (HR) motifs. Crystallographic studies show that the HR motifs fold into an HR1–HR2 six-helix bundle^34–37^. Further, the pre- and post-fusion structures of the S glycoprotein of mouse hepatitis virus (MHV) show the structural rearrangements of the MHV S2 subunit during membrane fusion^38,39^. However, detailed structural information about the SARS-CoV, MERS-CoV, and SARS-CoV-2 S2 fusion machineries is still required for further understanding of the mechanism of membrane fusion and the potential for developing broad-spectrum therapeutics against these human-infecting coronaviruses with serious pathogenicity.

Here we used cryo-electron microscopy (cryo-EM) to determine the structure of the post-fusion SARS-CoV S2 trimer. We present an analysis of the N-linked glycan shield masking the surface of the S2 machinery. Comparison with the structure of the pre-fusion SARS-CoV S glycoprotein reveals dramatic conformational changes of the SARS-CoV S2 machinery during membrane fusion, resembling that of MHV and other class I fusion proteins. Further, we found that the linker regions upstream of the HR2 motifs that are critical for the formation of the HR1–HR2 six-helix bundle bind more tightly along the central stem region than the MHV S glycoprotein, and that the HR1–HR2 six-helix bundle is more twisted leading to a less buried area between the HR1 and HR2 motifs. By mapping antibody and inhibitor targets within the conserved S2 subunit, we provide the structural basis for the development of vaccines and therapies that may suitable for treating a broad range of SARS-like coronaviruses.

## Results

### Post-fusion architecture of the SARS-CoV S2 fusion machinery

**Fig. 1:**
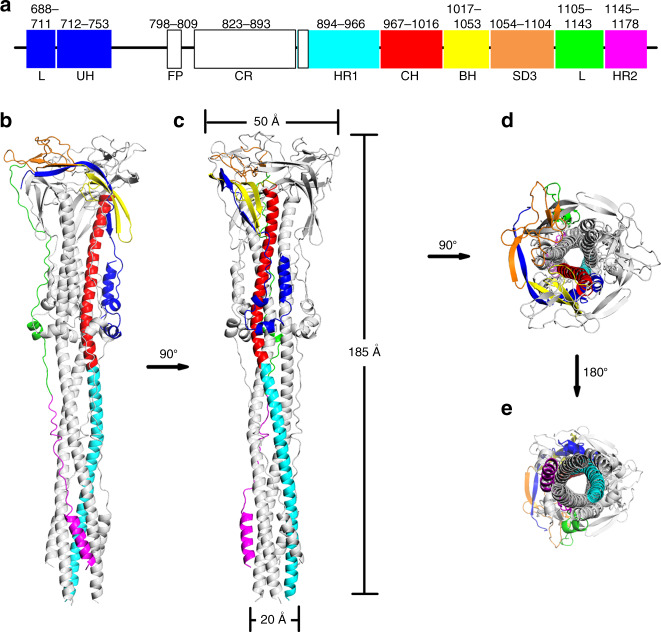
Overall structure of SARS-CoV S2 fusion machinery in post-fusion state. **a** Schematic diagram of the SARS-CoV S2 subunit ectodomain. The uncolored region represents the undetermined region in our structure. L linker region, UH the upstream helices, FP fusion peptide, CR connecting region, HR heptad repeat, CH central helix, BH β-hairpin, SD3 subdomain 3. **b**–**e** Cartoon representation of the SARS-CoV S2 trimer in post-fusion state colored as shown in a.

We produced the post-fusion SARS-CoV S2 subunit by subjecting the pre-fusion S ectodomain to trypsin digestion, receptor binding and low pH treatment, and determined the cryo-EM structure of the induced post-fusion SARS-CoV S2 trimer at a resolution of 3.9 Å (Supplementary Figs. 1 and 2). The final structure includes all domains and regions in the ectodomain of the S2 subunit (residues 688–1178), except for the fusion peptide (FP) and its downstream connecting-region (CR) motifs (residues 754–900). Similar to the structure of the post-fusion MHV S2 subunit generated by directly expressing only the S2 subunit ectodomain^39^, the overall structure of the SARS-CoV post-fusion S2 trimer adopts the shape of a baseball bat, 20–50 Å wide and 185 Å long (Fig. 1). The structure of the post-fusion SARS-CoV S2 fusion machinery comprises a long central helical bundle surrounded by short helices and β-sheets at the distal end of the membrane (Fig. 1). The HR1 and the central helix (CH) motifs fold into a continuous 31-turn α-helix. Three HR1-CH long helices intertwine with each other to form the stem region of the post-fusion S2 trimer with an outward orientation from the membrane side. The HR2 motif and its upstream linker region bind into the groove at the interface between the two HR1-CH helices of the neighboring protomers in an antiparallel manner, reminiscent of the six-helix bundle present in the crystal structures of the HR1 and HR2 motifs^34–37^. The upstream helix (UH), β-hairpin (BH) motif, and subdomain 3 (SD3) are located surrounding the C-terminus of the central HR1-CH helices, which only partially cover the top of the central helical region.

**Fig. 2:**
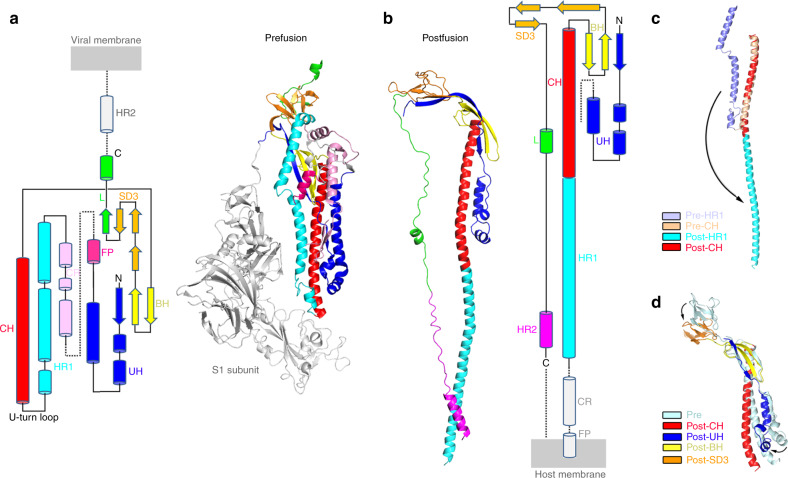
Structural and conformational changes of SARS-CoV S glycoprotein from pre-fusion to post-fusion state. **a** Topology and cartoon representation of SARS-CoV S2 subunit in pre-fusion state^20^ (PDB: 6NB6) colored as in Fig. 1a. The S1 subunit in the pre-fusion state is colored white. **b** Topology and cartoon representation of SARS-CoV S2 subunit in post-fusion state colored as in Fig. 1a. Unobserved regions in the structures are colored in light gray or presented as dashed lines in the topology diagram of (**a**, **b**). **c** Structural transition of the HR1 and CH motifs during membrane fusion. **d** Structural changes of the UH, BH, and SD3 during membrane fusion.

Transition from pre-fusion to post-fusion state triggers dramatic structural rearrangements and conformational changes of the SARS-CoV S glycoprotein (Fig. 2), resembling those of MHV S, influenza virus HA, paramyxovirus F and other class I viral fusion machineries^39–41^. The largest structural rearrangement lies in the HR1-CH helical region (Fig. 2c). In the pre-fusion state, the HR1 and CH regions comprise separate short helices, and the HR1 helices and the CH helix are connected through a U-turn loop, which places the two motifs into an antiparallel orientation. During membrane fusion, the HR1 motif turns over to form a continuous stem helix together with the CH motif pointing toward the target membrane, which makes the post-fusion SARS-CoV S2 fusion machinery extend ~80 Å longer than the pre-fusion S2 trimer. Note that all flexible connecting loops from the HR1 and CH motifs in the pre-fusion state rearrange into the long single stem helix in the post-fusion state. Such an onward helical extension contributes to the ejection of the FP. The UH, BH, and SD3 regions retain their tertiary structures in the pre-fusion state at the distal end of the target membrane, although the UH and SD3 regions slightly rotate relative to the CH helix, making the quaternary structure more compact (Fig. 2d and Supplementary Fig. 3a–c). As described in the post-fusion MHV S2 structure^39^, four highly conserved disulfide bonds (C720–C742, C725–C731, C1014–C1025, and C1064–C1108) are identified within the post-fusion SARS-CoV S2 trimer, which also exist in the pre-fusion state, and are located exactly around the UH, BH, and SD3 regions which are more stable than other motifs during membrane fusion (Supplementary Fig. 3). These observations suggest that these disulfide bonds, particularly the linkage between domains, help to maintain the structure of the S2 subunit during the dramatic transition that triggers membrane fusion.

### The linker upstream of HR2 is critical for membrane fusion

**Fig. 3:**
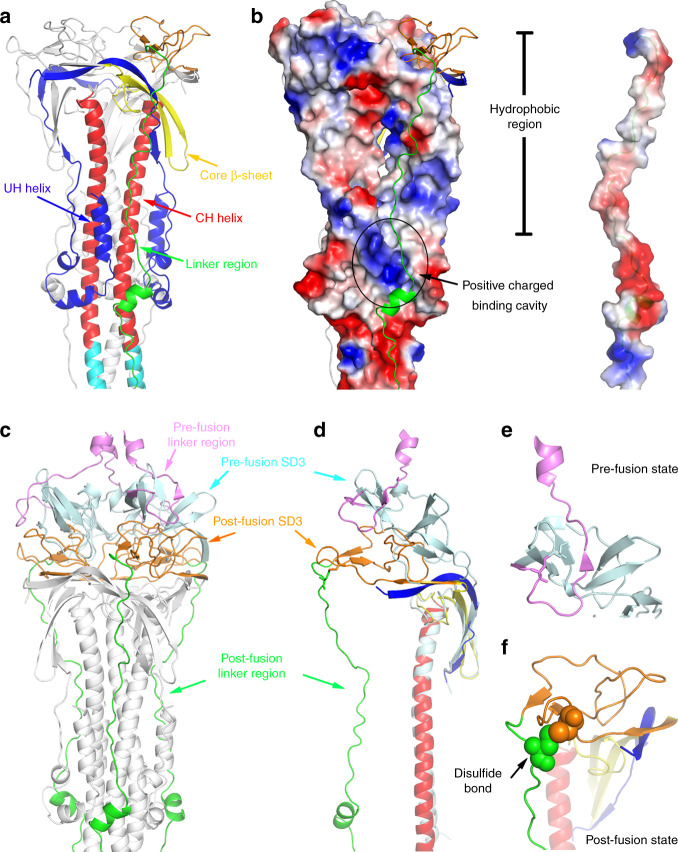
The linker region upstream of HR2 motif important for membrane fusion. **a** Binding of the linker region upstream of HR2 motif along the central helix colored as in Fig. 1a. **b** The electrostatic surface representation of the linker region upstream of HR2 motif binding area. Red represents negative charge, and blue represents positive charge. **c** Overall structural changes of the linker region upstream of the HR2 motif during membrane fusion. The linker regions in pre-fusion^20^ (PDB: 6NB6) and post-fusion states are shown as cartoons colored in violet and green, respectively. The SD3 domains in pre-fusion^20^ (PDB: 6NB6) and post-fusion states are shown as cartoons colored in pale cyan and orange, respectively. **d** Structural changes of the linker region upstream of the HR2 motif during membrane fusion represented in single chain mode. The cartoons are colored as in (**c**). **e** Structural details of the linker region and the SD3 domain from the pre-fusion SARS-CoV^20^ (PDB: 6NB6) colored as in (**c**). **f** Structural details of the linker region and the SD3 domain from the post-fusion SARS-CoV colored as in (**c**). The disulfide bond is shown as spheres.

The linker region upstream of the HR2 motif resolved in our post-fusion structure is partially visible in the structures of pre-fusion SARS-CoV and MERS-CoV S glycoproteins^20,29,33^, although could be almost completely observed in the structure of the pre-fusion human coronavirus NL63 (HCoV-NL63) S trimer^42^. In the pre-fusion state, a β-strand from the pre-fusion linker region forms a β-sheet with the SD3 domain so that the upstream linker region is well folded and retained adjacent to the SD3 domain (Fig. 3c–e). In contrast, in the post-fusion state, the linker region no longer folds into the β-sheet with the SD3 domain, but is turned over to the opposite direction and rearranged into an extended structure mainly assembled by loops that bind along the axis of the stem helices from adjacent protomers (Fig. 3). The N-terminus of the linker region is stabilized by interaction with the hydrophobic surface of the core β-sheet and the subsequent hydrophobic groove formed by the UH and CH helices (Fig. 3b). The C-terminal region binds to a positively charged cavity through its acidic amino acid enriched region (residues 1128–1133) (Fig. 3b). This observation indicates that the linker region upstream of the HR2 motif undergoes large structural rearrangements during membrane fusion, and correct refolding of this linker region is important for the formation of the central HR1–HR2 six-helix bundle, which brings the viral membrane close to the host membrane at the late stage in the fusion transition. Thus the linker region upstream of HR2 may be a potential therapeutic site for drug designation and antibody development to defense against coronavirus infection.

The disulfide bond (C1064–C1108) between the linker region upstream of the HR2 motif and the SD3 domain helps stabilize the linker region during the fusion transition. However, conservation analysis shows that while the other three disulfide bonds are completely conserved between SARS-CoV, SARS-CoV-2, MERS-CoV, and MHV, the disulfide linkage between the linker region upstream of HR2 motif and the SD3 domain does not exist in the MERS-CoV S glycoprotein, because the residue corresponding to C1108 in the linker region is replaced by a glutamine residue (Supplementary Fig. 3d). Considering that this disulfide linkage is the last contact between the β-sheet region from SD3 and the N-terminal end of the linker region in the post-fusion structure, this linkage as well as the rotation of the SD3 domain contribute to positioning the linker region in the correct direction along the axis of the stem helix toward the target membrane, and thus benefits subsequent binding of the linker region and HR2 motif into the groove formed by the HR1-CH helices (Fig. 3f).

### Comparison of coronaviruses fusion machineries

**Fig. 4:**
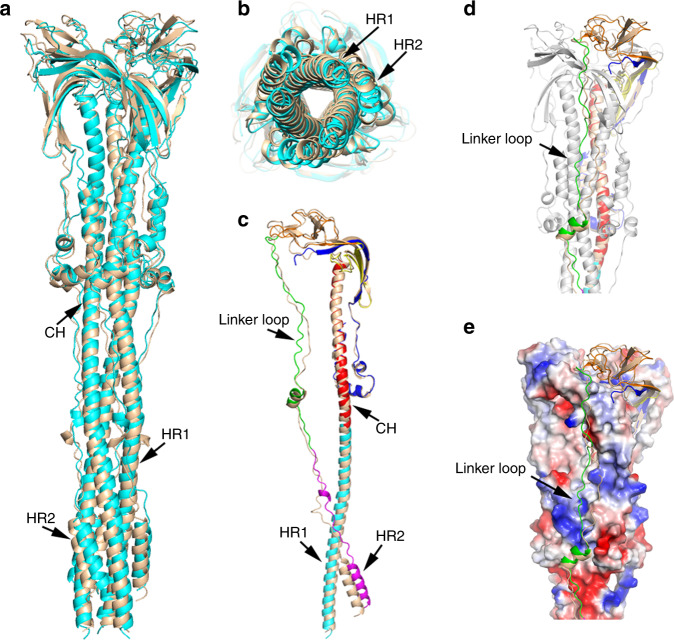
Comparison of SARS-CoV and MHV S2 structures in post-fusion state. **a**, **b** Cartoon representation of the comparison of post-fusion S2 overall structures from SARS-CoV (cyan) and MHV^39^ (PDB: 6B3O, wheat). **c** Superposition of SARS-CoV (colored as Fig. 1a) and MHV^39^ (PDB: 6B3O, wheat) post-fusion S2 subunits in single chain mode. **d**, **e** Structural comparison of the linker region upstream of HR2 motif from SARS-CoV (green) and MHV^39^ (PDB: 6B3O, wheat) shown as cartoon and electrostatic surface. Red represents negative charge, and blue represents positive charge.

Consistent with the 40% amino acid sequence identity between SARS-CoV and MHV S2 subunits, the post-fusion structures of SARS-CoV and MHV S2 trimers adopt a similar architecture with an overall root mean square deviation of 2.43 Å (Fig. 4). Structure superposition analysis reveals that the SARS-CoV HR2 helices twist ~4 Å around the central HR1 helices compared with that of MHV, while the HR1-CH helices slightly rotate (Fig. 4a–c). This rotation forces the SARS-CoV HR2 motifs (residues 1145–1178) to be less buried by the groove assembled from the HR1 helices, with a buried surface area of 5162 Å^2^ compared with 5780 Å^2^ between the MHV HR2 motifs (residues 1215–1248) and the grooves. In contrast, the SARS-CoV linker region (residues 1105–1143) upstream of the HR2 motif binds tightly along the central stem helix, while the MHV linker region (residues 1174–1212) slightly shifts outward from the binding groove (Fig. 4c–e), which makes the accessible surface area of the MHV linker regions less buried for ~345 Å^2^. These structural differences suggest that the SARS-CoV linker region upstream of the HR2 motif may contribute to the formation of the six-helix bundle exceeding that in MHV.

Superposition of the crystal structures of the HR1–HR2 six-helix bundles from MHV and SARS-CoV shows that the SARS-CoV HR2 helix in the crystal structure is rotated ~1.5–2.5 Å around the stem helices compared with that of MHV^35^, which is slightly less twisted than the HR2 motif in our S2 trimer structure (Supplementary Fig. 4). The absence of restriction and stabilization of other regions besides the HR1 and HR2 motifs in the crystal structure may account for these differences. The three CH helical regions that form continuous α-helices with the HR1 motifs are more intertwined in our post-fusion S2 machinery compared with the MHV CH helices (Fig. 4a–c), and thus initiating the slight rotation of the refolded HR1 helices (Fig. 4a–c) and further leading to twist of the HR2 motifs. However, when we compared the crystal structures of the HR1–HR2 helices of MERS-CoV and MHV, we found that their structures adopt similar architectures without rotation (Supplementary Fig. 4c). Hence, these observations indicate that the rotation of the HR2 motif may represent a unique structural feature of SARS-CoV and SARS-CoV related coronaviruses, such as the SARS-CoV-2 that shares 91% sequence identity with the SARS-CoV S2 subunit. We predicted the post-fusion structure of SARS-CoV-2 glycoprotein through SWISS-MODEL^43^ using the post-fusion SARS-CoV S2 trimer as template (Supplementary Fig. 5).

### The glycan shield of the post-fusion SARS-CoV S2 trimer

**Fig. 5:**
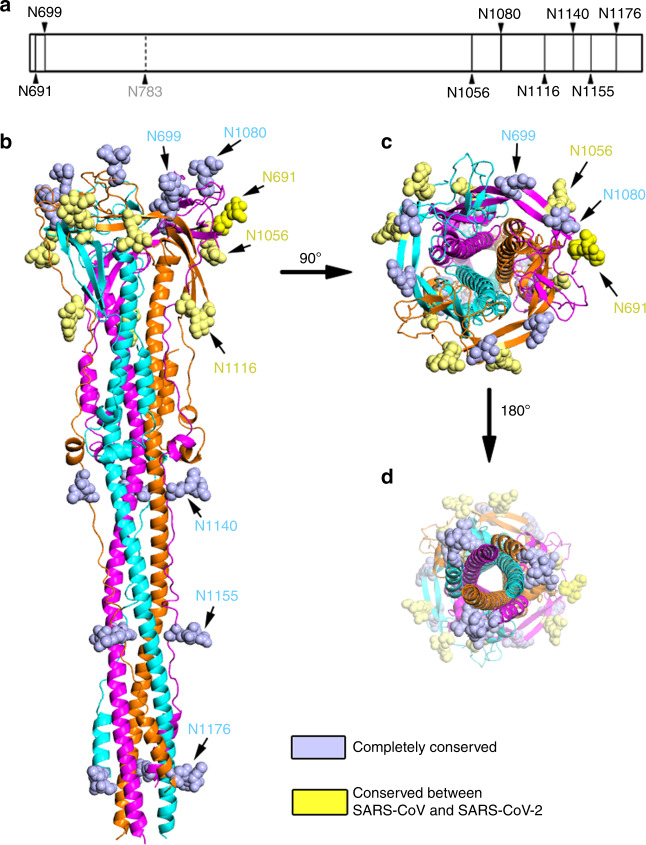
Glycan shield of SARS-CoV S2 fusion machinery in post-fusion state. **a** Schematic diagram of the N-linked glycosylate modification sites of SARS-CoV S2 subunit. The visible N-linked glycosylation sites are shown in solid lines and the invisible N-linked glycosylation site is shown in dashed line. **b**–**d** The distribution of the glycan shield within the S2 subunit shown in side view (**b**), top view (**c**) and bottom view (**d**). The highly conserved glycosylation sites among coronavirus are shown as light blue spheres. The completely conserved glycosylation sites between SARS-CoV and SARS-CoV-2 are shown as yellow spheres.

The S glycoprotein is the major antigenic determinant exposed on the viral surface. Coronavirus deploys a large number of N-linked glycans that cover the surface of the S glycoprotein to limit its accessibility to neutralizing antibodies and evade attack by the host immune system. Our structure identified 8 out of 9 putative N-linked glycans, which reveal the distribution of the glycan shield covering the surface of the post-fusion SARS-CoV S2 trimer (Fig. 5). Four N-linked glycosylation sites (N691, N699, N1056, and N1080) are located in the relatively stable β-sheet enriched region surrounding the C-terminus of the central stem helices. The other four glycosylation sites (N1116, N1140, N1155, and N1176) reside within the HR2 motif and its upstream linker region. Conservation analysis shows that 5 N-linked glycosylation sites (N699, N1080, N1140, N1155, and N1176) are completely conserved among SARS-CoV, SARS-CoV-2, MERS-CoV, and MHV, whereas three N-linked glycosylation sites (N691, N1056, and N1116) are only conserved between SARS-CoV and SARS-CoV-2 (Supplementary Fig. 6). These glycans mask the accessible surface of the corresponding regions during transition from pre-fusion to post-fusion, which may protect the S2 subunit from antibody recognition, reminiscent of the glycans modifying HCoV-NL63 S^42^, and HIV-1 Env^44^ that contributes to immune evasion by masking the protein surface. The enrichment of glycosylation sites in the HR2 motif and its upstream linker region indicates the importance of these regions for S2-mediated membrane fusion.

### Potential therapeutic targets within the S2 fusion machinery

**Fig. 6:**
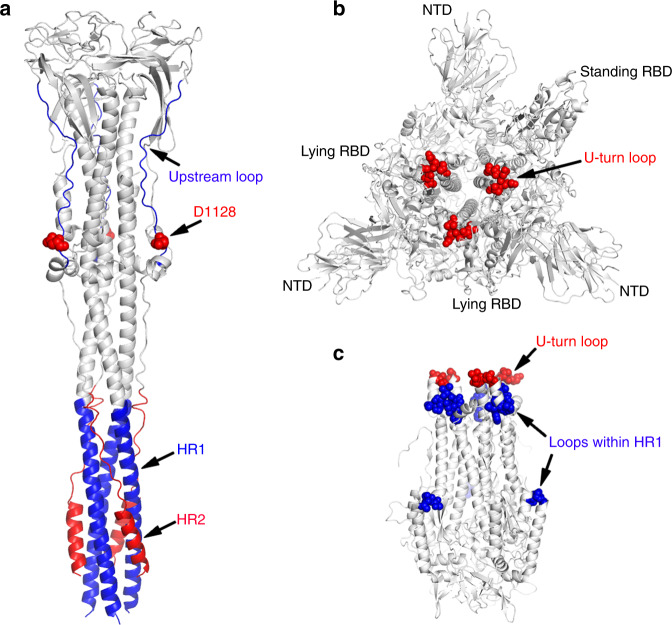
Potential therapeutic targets within SARS-CoV S2 subunit. **a** Cartoon representation of the linker loop region upstream of the HR2 motif (blue loop), the HR1 motif (blue helix) and the HR2 motif (red). The residue D1128 is shown as spheres colored in red. **b** The U-turn loop connecting the HR1 and CH motif in the pre-fusion SARS-CoV S trimer^27^ (PDB: 5X 5B). The pre-fusion SARS-CoV structure is shown as cartoons colored in white. The U-turn loop is presented as spheres colored in red. **c** The U-turn loop and other loop regions within the HR1 and CH motifs in the pre-fusion S2 subunit^27^ (PDB: 5X 5B). The U-turn loop is shown as spheres colored in red and the other loop regions are shown as spheres colored in blue.

The therapeutic targets in coronavirus S glycoprotein mainly reside in the RBD region of the S1 subunit^20,21^ and in the HR1–HR2 six-helix bundle of the S2 subunit^22–24^. Binding of antibodies and inhibitors to the RBD can prevent viral entry by inhibiting the association of the S glycoprotein with the receptor on the surface of target cells. Antibodies and inhibitors against S2 subunit can inhibit virus infection by blocking S2-meditated membrane fusion. Many peptide inhibitors of the S2 subunit are derived from the sequences of the HR1 or HR2 motifs, which prevent the fusion core formation by interacting with the corresponding HR2 or HR1 motif in the S2 fusion machinery^45,46^. The major target regions of peptide inhibitors are residues 902–947 of the HR1 motif and residues 1149–1186 of the HR2 motif^45,46^, which map to the hydrophobic HR1–HR2 interacting groove in the post-fusion S2 trimer (Fig. 6a). Moreover, some antibodies also target the HR1–HR2 interface of SARS-CoV S2 subunit^22,26^. Besides the therapeutics toward HR1–HR2 binding groove, antibodies against other parts of the S2 subunit are also reported to block the viral entry mediated by S glycoprotein^22,23^. A previous study developed two groups of monoclonal antibodies (mAbs) targeting the region upstream of the HR2 motif of SARS-CoV, including the C-terminus of the SD3 domain and the HR2 upstream linker region^22^. Further study found that one of the antibodies targeting the epitope within the residues 1111–1130 of the HR2 upstream linker region (Fig. 6a), named mAb 1A9, interacts with D1128 of the SARS-CoV S2 subunit, and D1128A substitution enables the virus to escape the inhibition by mAb 1A9^23^. D1128 is located within the acidic amino acids enriched region (residues 1126–1133) of the linker loop upstream from the HR2 motif, which binds to the positively charged cavity in the main body as shown in our post-fusion SARS-CoV S2 structure (Figs. 3b and 6a). It is possible that mAb 1A9 binds to D1128 and its adjacent region, which prevents the linker loop from binding along the CH, thus blocking the association of the HR2 motif with the HR1 helices in the late stage of membrane fusion. Interestingly, the mAb 1A9 confers cross-protection against viral entry mediated by the S glycoproteins of human SARS-CoV, civet SARS-CoV and bat SL-CoVs^23^. Note that residue D1128 and its adjacent sequences within the upstream linker loop are completely conserved between SARS-CoV and SARS-CoV-2 (Supplementary Fig. 6). Moreover, certain mAbs targeting the highly conserved HR1–HR2 interacting region of SARS-CoV could also inhibit viral entry mediated by S proteins of clinical isolates with different RBD sequences^26^. Further, the HR2 motif is completely conserved between SARS-CoV and SARS-CoV-2 although the HR1 motif is relatively variable (Supplementary Fig. 6). These results indicate that the conserved regions of the HR2 motif and its upstream linker loop, which are both required for the formation of the six-helix bundle, as shown in our post-fusion S2 structure, are important potential targets for broad-spectrum vaccines and drugs development against SARS-like coronaviruses.

## Discussion

Here we present the structure of the induced post-fusion SARS-CoV S2 machinery, which undergoes large scale of structural rearrangements and conformational changes compared with its pre-fusion structure. Structural analysis indicates that the linker region upstream of the HR2 motif that binds along the stem helix plays an important role in the subsequent formation of the HR1–HR2 six-helix bundle, which is critical for viral entry mediated by class I fusion proteins. The fusion mechanism revealed by the SARS-CoV S2 trimer resembles that of other class I fusion glycoproteins^47^. According to the structures of the pre- and post-fusion SARS-CoV S glycoproteins, we propose a model of SARS-CoV fusion process (Supplementary Fig. 7). After cleavage at the boundary between the S1 and S2 subunits, most of the S glycoproteins are retained in the pre-fusion trimeric architecture. Further binding to the receptor on the host membrane surface by the standing RBD and cleavage of the S2′ site by host protease accelerate the release of the S1 subunit. Then the helical regions in the remaining S2 trimer are refolded, which mediates the insertion of the FP into the target membrane. The UH, BH, and SD3 regions retain their structures adjacent to the C-terminus of the CH helix. Next, the linker region upstream of the HR2 motif is released and extends along the axis of the stem helices. Thus the downstream HR2 motif gets close to the HR1 helix and forms the six-helix bundle at the proximal side of the host membrane, which mediates fusion between the viral and host membranes. Considering the amino acid sequence conservation of the S2 subunit among coronaviruses, it is possible that all the coronaviruses may mediate vial-host membrane fusion through a similar mechanism.

From 2003 to nowadays, the highly infectious human pathogens SARS-CoV, MERS-CoV, and SARS-CoV-2 are responsible for severe global health emergencies^15^. Considering the potential risk of the spread of these or unknown coronaviruses in the future, as well as the relatively high mutation rates of RNA viral genomes during replication, development of broad-spectrum antiviral vaccines and therapeutics are required to prevent a wide range of coronaviruses infection. Because of its important role in mediating receptor recognition and viral entry, the S glycoprotein, the major antigen on the surface of coronaviruses, is the key determinant required for their efficient transmission among species. A majority of antibodies and inhibitors target the RBD region of the S1 subunit to inhibit virus infection by blocking the interaction with the host receptor. However, applications of RBD-specific antibodies are limited by the variability of RBD amino acid sequences, which is consistent with different receptor usage by different coronaviruses. Moreover, mutations in the RBD region may not affect viral entry but instead impair the efficacy of the RBD-specific treatment. For example, although four out of five residues critical for receptor binding are replaced in the RBD domain of SARS-CoV-2 S glycoprotein, the SARS-CoV-2 RBD retains strong binding affinity to the SARS-CoV receptor ACE2 and efficiently infects human cells^48^. Recent studies of the pre-fusion SARS-CoV-2 S glycoprotein^30,31^ and the structures of SARS-CoV-2 RBD bound to ACE2^49–52^ also demonstrate the binding ability of SARS-CoV-2 RBD with ACE2. In contrast, therapeutics developed based on the S2 subunit may be effective against a wide range of coronaviruses, because the amino acid sequence of the S2 subunit is more conserved than that of the S1 subunit due to their critical roles in mediating the complex process of membrane fusion (Fig. S7). For example, the S1 subunit of the newly identified SARS-CoV-2 shares 64% amino acid sequence identity with the SARS-CoV S1 subunit compared with 91% for the S2 subunits. Note that certain SARS-CoV S2-specific antibodies that recognize the interface groove of the HR1–HR2 six-helix bundle and the linker region upstream of the HR2 motif neutralize a broader spectrum of SARS-CoV clinical isolates or variants^23,26^. Moreover, the peptide inhibitor P9, which binds the influenza HA glycoprotein and the MERS-CoV S2 subunit, shows antiviral ability against a broad range of respiratory viruses, including influenza viruses, MERS-CoV and SARS-CoV^53^. Targeting other conserved regions within the S2 subunit may also contribute to the development of broadly reactive virus therapeutics. For example, the U-turn loop connecting the HR1 and CH motifs in the pre-fusion state, which refolds into the continuous HR1-CH helix during membrane fusion as shown in our structure, are accessible in the pre-fusion S trimer when the variable RBD domain is in the standing state (Fig. 6b). This U-turn loop and its adjacent regions are completely conserved between SARS-CoV and SARS-CoV-2 (Supplementary Fig. 6). Moreover, other loop regions within the HR1 and CH motifs, which are highly conserved as well, are completely exposed after dissociation of the S1 subunits (Fig. 6c and Supplementary Fig. 6). Small molecule inhibitors and neutralizing antibodies against these conserved loops may also inhibit virus entry by preventing the formation of the HR1–CH stem helices of a broader range of coronaviruses. Thus, we propose that the conserved regions within the S2 subunit, including the HR2 motif, the linker region upstream of the HR2 motif, and the loop regions of the HR1 and CH motifs are important potential targets for developing broad-spectrum vaccines and therapeutics. Moreover, we describe most of the glycosylate modifications of the SARS-CoV S2 fusion machinery, which may contribute to immune evasion by concealing the protein surface and are completely conserved between SARS-CoV and SARS-CoV-2. Thus our structure provides more critical structural information that will likely facilitate the development of effective antibodies and inhibitors.

In summary, the structure of the post-fusion SARS-CoV S2 trimer determined here, which reveals the host-cell fusion mechanism of coronaviruses and the glycan shield that masks the protein surface, may help guide the development of antibodies and drugs against SARS-CoV and other SARS-like coronaviruses, particularly the newly discovered SARS-CoV-2, which is still spreading worldwide.

## Methods

### Protein expression and purification

Sequences encoding the ectodomain of the SARS-CoV spike protein (UniProtKB accession P59594, residues 14–1,193) were optimized for synthesis and inserted into the baculovirus transfer vector pFastbac1 (Invitrogen) with an N-terminal gp67 signal peptide, a C-terminal thrombin cleavage site followed by a T4 fibritin trimerization domain and a His_6_-tag. Transfection and virus amplification were performed using Sf9 cells, and Hi5 cells were used to express the recombinant proteins using the Bac-to-Bac system. The recombinant proteins were harvested from cell culture medium and sequentially purified using Ni-NTA and Superdex 200 columns (GE Healthcare).

The sequence encoding the extracellular domain of human ACE2 (UniProtKB accession Q9BYF1, residues 19–615) with an N-terminal gp67 signal peptide and a C-terminal His_6_-tag was optimized for synthesis and subcloned into the baculovirus transfer vector pFastbac1 (Invitrogen). Transfection and virus amplification were performed using Sf9 cells, and Hi5 cells were used to express the recombinant proteins using the Bac-to-Bac system. The proteins were harvested from cell culture medium and purified using a Ni-NTA column. Fractions containing ACE2 were collected and applied directly to a QHP column (GE Healthcare), and then eluted with linear gradient of 0.15–1.0 M NaCl in 20 mM Tris buffer (pH 8.0). Fractions containing ACE2 were then purified using a Superdex 200 column with a buffer containing 20 mM Tris–HCl (pH8.0) and 150 mM NaCl.

### Preparation of the S2 subunit in post-fusion state

Purified S proteins were incubated with trypsin (Sigma-Aldrich, trypsin mixed with S protein at a mass ratio of 1:100) and thrombin (Sigma-Aldrich, 3 units/mg S protein) overnight at room temperature to cleave at the S1/S2 boundary and remove the C-terminal trimerization domain and the His_6_-tag. Size exclusion chromatography (Superdex 200 column) was performed to purify the cleaved product. The S1 and S2 subunits still formed stable complexes according to the results of gel-filtration chromatography and SDS-PAGE analysis. Then the concentrated sample was mixed with 1/10 volume of 1 M sodium citrate pH 5.5, CaCl_2_ with a final concentration of 2 mM, and human receptor ACE2 (S protein mixed with ACE2 at a molar ratio of 1:20), followed by incubation at room temperature for 16 h. The mixture was purified by gel-filtration chromatography using a Superdex 200 column with a buffer containing 20 mM Tris pH7.5 and 150 mM NaCl, and then analyzed using SDS-PAGE.

### Cryo-EM data collection and processing

Purified protein samples with a concentration of 0.1 mg/ml were placed on a glow-discharged holey carbon grid (Quantifol, Au 400 mesh, R 0.6/1.0). After blotting with filter paper for 4 s, the grid was flash plunged into liquid ethane using an automatic plunge freezer (Leica EM GP2) operated at 10 °C and 75% humidity. Cryo-EM single-particle data collection was performed through our newly developed beam-image shift data collection method^54^ using a 200 kV FEI Tecnai Arctica microscope equipped with a K2 camera (Gatan). Images were recorded at a defocus range of −1.8 to −2.2 μm with a pixel size of 1.0 Å. The exposure time was 5.12 s, with a total exposure dose of ~50 electrons per Å^2^ over 32 frames.

Each image stack was subjected to motion correction using MotionCor2^55^ before further data processing and resulted in an averaged micrograph. The parameter of the contrast transfer function on each micrograph was determined by the program CTFFIND4^56^. All subsequent processing steps were executed using RELION 2.1^57^ (Fig. S2). A subset of protein particles was manually picked and processed with reference-free 2D classification. Three representative 2D class averaged images were selected as references for automatic particle picking of the entire dataset, which resulted in a total of 343,042 particles. Then the particles were processed by reference-free 2D classification. After two rounds of 2D classification, 69,792 particles were selected for further 3D classification, which were classified into five classes using the MHV S2 post-fusion trimer density map^39^ as an initial model. 40,600 particles from class 4 were subjected to further 3D auto-refinement with C3 symmetry, yielding a 3.9 Å density map estimated according to the gold-standard Fourier shell correlation with the 0.143 criterion. The local resolution of the final density map was calculated using ResMap^58^.

### Model building and refinement

For model building of the post-fusion SARS-CoV S2 trimer, the structure of SARS-CoV S2 subunit was predicted by Phyre2^59^ using the post-fusion MHV S2 trimer (PDB: 6B3O)^39^ as template. Then, the predicted structure was fitted into the 3.9 Å resolution map using UCSF Chimera^60^ and manually rebuilt in Coot^61^. The model was further improved through cycles of real-space refinement in Phenix^62^ with geometric and secondary structure restraints, and subsequent manual corrections by Coot were carried out iteratively. The refinement statistics of the model generated in Phenix are summarized in Supplementary Table 1. Figures representing the structural features were prepared with UCSF Chimera^60^ and PyMOL (http://pymol.org).

### Reporting summary

Further information on research design is available in the Nature Research Reporting Summary linked to this article.

## Supplementary information

Supplementary Information

Reporting Summary

## Data Availability

The data that support this study are available from the corresponding authors upon reasonable request. The cryo-EM density map of post-fusion SARS-CoV S2 trimer was deposited in the Electron Microscopy Data Bank (EMDB) with accession code EMD-30072. The corresponding model was deposited in the Protein Data Bank (PDB) with accession code 6M3W.
