# Supplementary material for: Cryo-EM analysis of the post-fusion structure of the SARS-CoV spike glycoprotein
Source: Nat Commun. 2020 Jul 17;11:3618. doi: 10.1038/s41467-020-17371-6 (PMC7367865; doi:10.1038/s41467-020-17371-6)
Supplement: Supplementary file 1 — Supplementary Information [file 41467_2020_17371_MOESM1_ESM.pdf]

Supplementary Information

Cryo-EM analysis of the post-fusion structure of the SARS-CoV spike glycoprotein

Xiaoyi Fan[1,2,4], Duanfang Cao[1,4,*], Lingfei Kong[1], Xinzheng Zhang[1,2,3,*]

Affiliations:

[1]National Laboratory of Biomacromolecules, CAS Center for Excellence in Biomacromolecules, Institute of Biophysics, Chinese Academy of Sciences, Beijing 100101, P.R. China.

[2]University of Chinese Academy of Sciences, Beijing 100049, P.R. China.

[3]Center for Biological Imaging, CAS Center for Excellence in Biomacromolecules, Institute of Biophysics, Chinese Academy of Sciences, Beijing 100101, P.R. China.

[4]These authors contributed equally to this work.

[*]Correspondence to : caodf@ibp.ac.cn (D.C.); xzzhang@ibp.ac.cn (X.Z.)

Supplementary Information includes：

- Supplementary figures 1-7
- Supplementary table 1
- Supplementary references

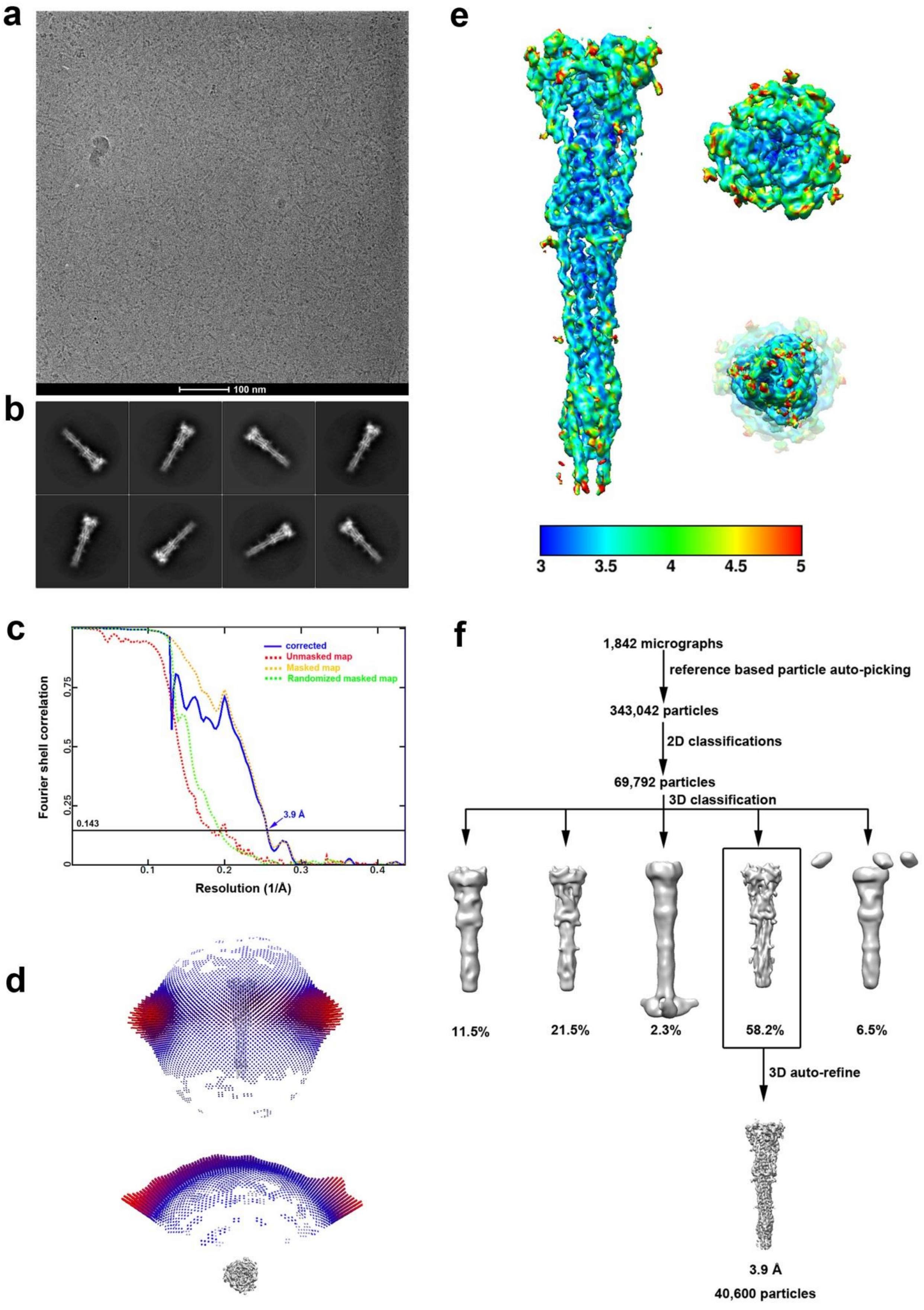
a
100 nm
b
c
Fourier shell correlation
Resolution (1/Å)
corrected
Unmasked map
Masked map
Randomized masked map
0.143
3.9 Å
d
e
3
3.5
4
4.5
5
f
1,842 micrographs
reference based particle auto-picking
343,042 particles
2D classifications
69,792 particles
3D classification
11.5%
21.5%
2.3%
58.2%
6.5%
3D auto-refine
3.9 Å
40,600 particles

Supplementary Figure 1. Cryo-EM analysis of SARS-CoV S2 machinery in post-fusion state. (**a**). Representative cryo-EM micrograph of post-fusion SARS-CoV S2 machinery. (**b**) Representative 2D class averages. (**c**) Gold-standard Fourier shell correlation (FSC) curves of the post-fusion SARS-CoV S2 trimer. (**d**) Angular distribution of the particles in the final 3D-refinement shown from side view (above) and top view (below). (**e**) Local resolution maps of the post-fusion SARS-CoV S2 trimer in side view (left) and top view (right). (**f**) Cryo-EM data processing workflow.

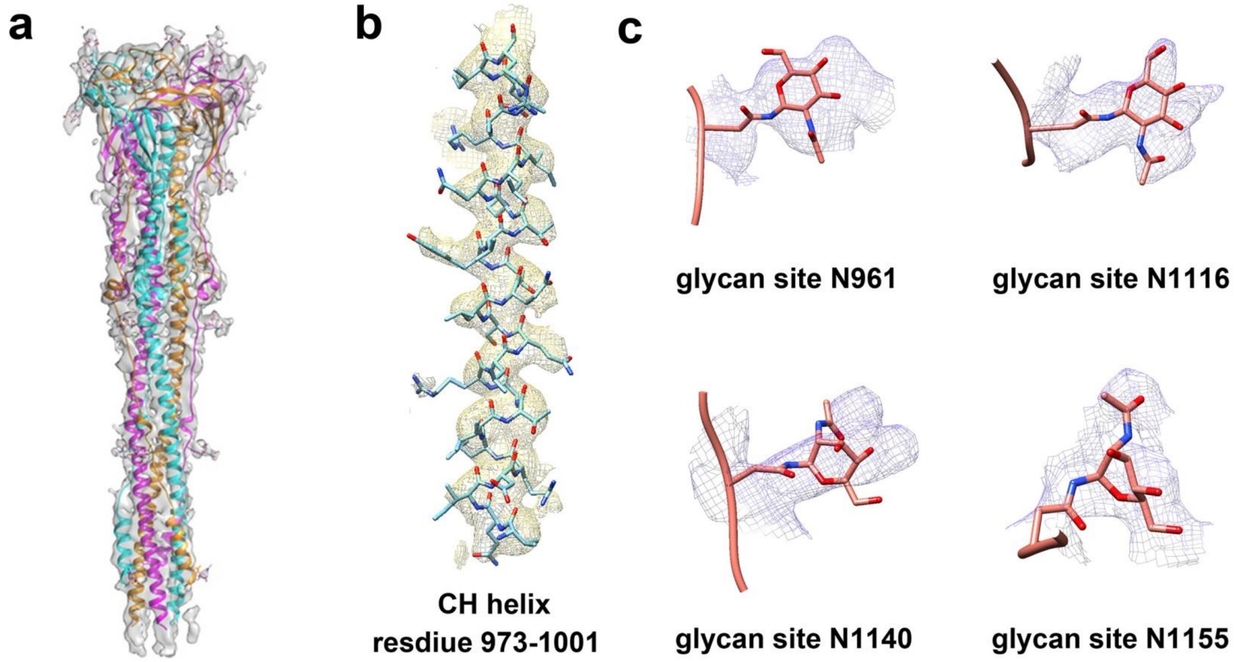

Supplementary Figure 2. Structural validation of the post-fusion SARS-CoV S2 trimer. (**a**) overall structure of the post-fusion SARS-CoV S2 trimer fit in the cryo-EM density map. (**b-c**) representative cryo-EM densities of the CH helix (b) and the glycosylate modifications (c).

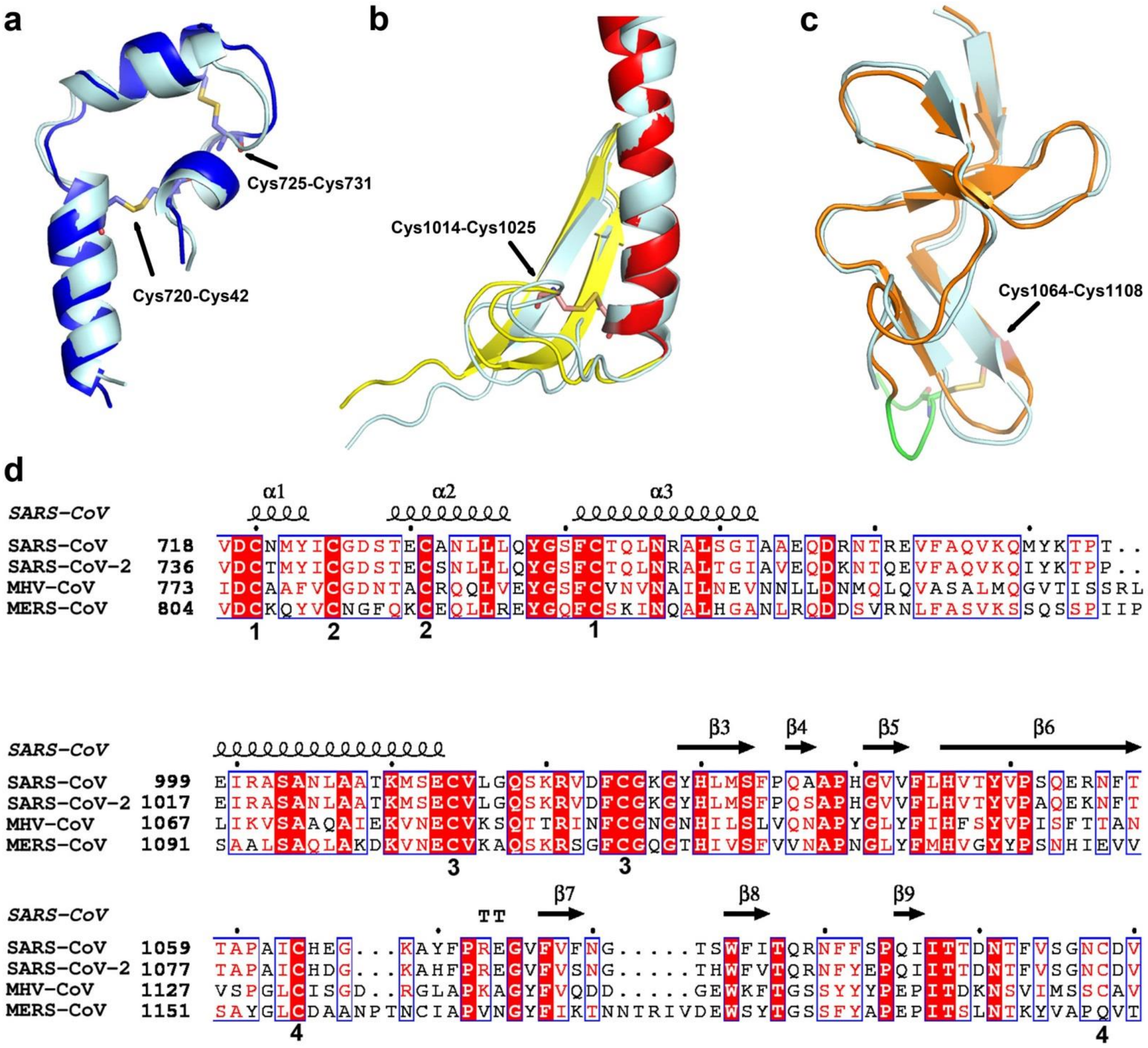

Supplementary Figure 3. The relative stable UH, BH and SD3 motifs during fusion transition. (**a**) Comparison of the UH helices in pre-fusion state (pale cyan) and post-fusion state (blue). (**b**) Comparison of the BH region in pre-fusion state (pale cyan) and post-fusion state (BH, yellow; CH, red). (**c**) Comparison of the SD3 region in pre-fusion state (pale cyan) and post-fusion state (orange). The disulfide bonds in a-c are shown as sticks. (**d**) Sequence conservation analysis of SARS-CoV, SARS-CoV-2, MHV-CoV and MERS-CoV representing the highly conserved disulfide bonds. The numbers of the four disulfide bonds are labeled under the sequences.

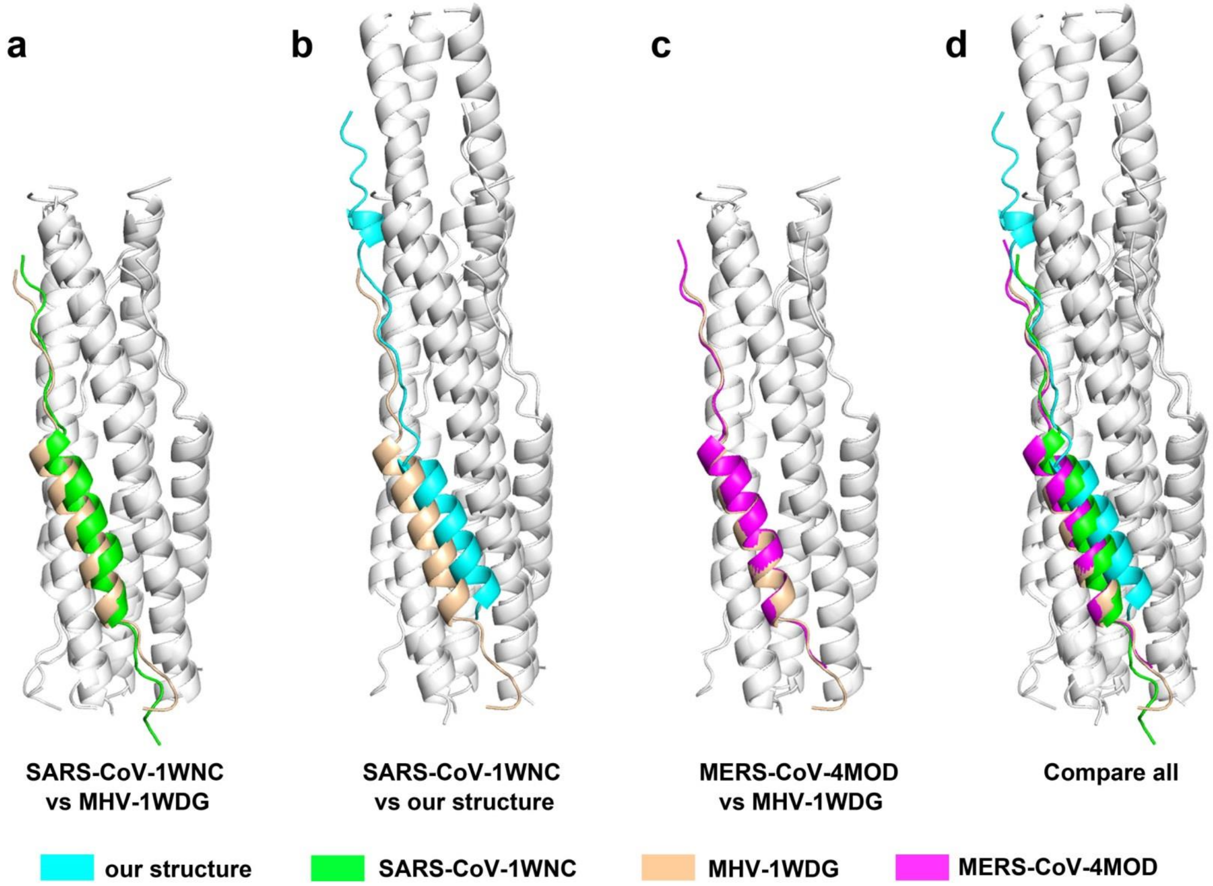

Supplementary Figure 4. Superposition of the HR1-HR2 six-helix bundle structures. (**a**) Comparison of the HR1-HR2 bundle from SARS-CoV crystal structure[1] (PDB:1WNC) and MHV crystal structure[2] (PDB:1WDG). (**b**) Comparison of the HR1-HR2 bundle from our post-fusion SARS-CoV S2 trimer and MHV crystal structure[2] (PDB: 1WDG). (**c**) Comparison of the HR1-HR2 bundle from MERS-CoV crystal structure[3] (PDB: 4MOD) and the MHV crystal structure[2] (PDB: 1WDG). (**d**) Comparison of the HR1-HR2 bundle of the structures all above. The HR2 motifs are colored in green for SARS-CoV crystal structure, wheat for MHV crystal structure, magenta for MERS-CoV crystal structure and cyan for our S2 structure.

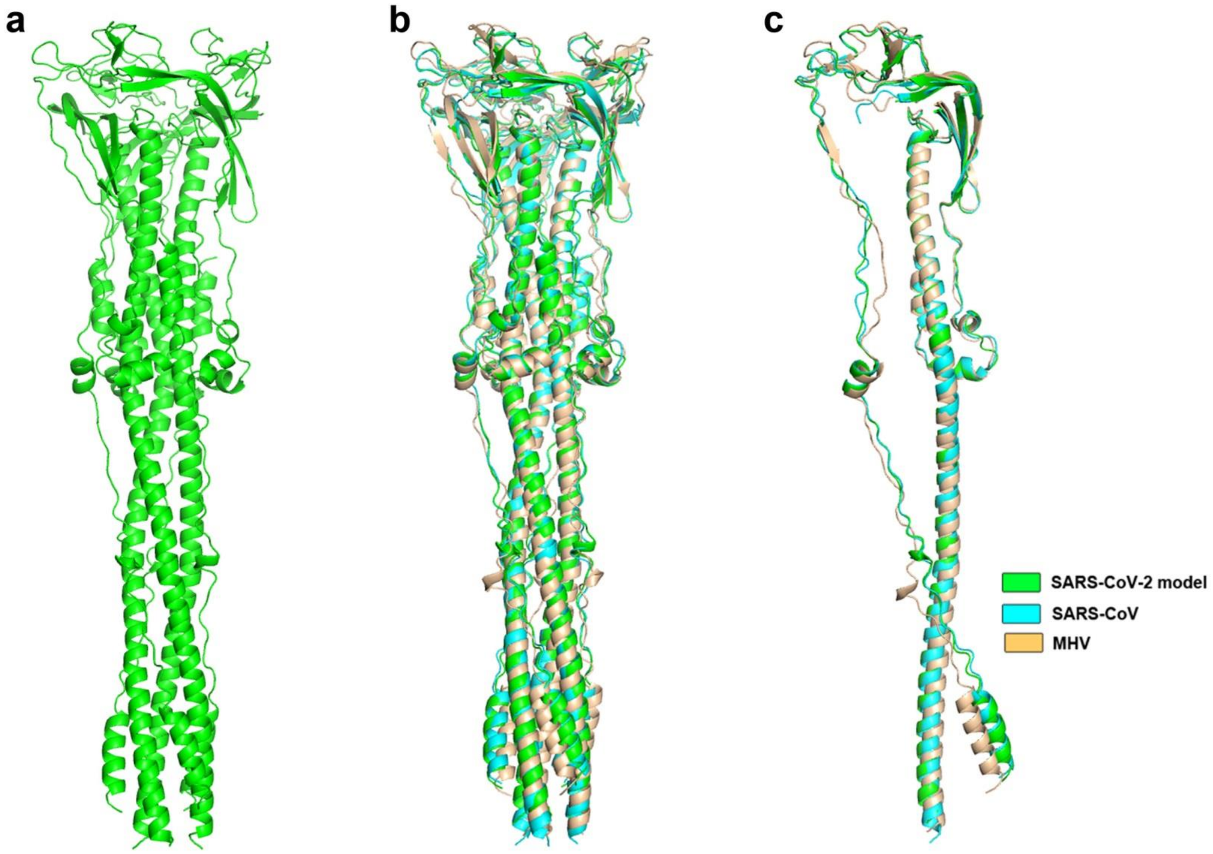

Supplementary Figure 5. Comparison of the post-fusion S2 structures from SARS-CoV and SARS-CoV-2. (**a**) Cartoon representation of the predict SARS-CoV-2 S2 trimer in post-fusion state. (**b**) Superposition of the SARS-CoV S2 trimer (cyan), MHV S2 trimer[4] (PDB: 6B3O, wheat) and predict SARS-CoV-2 S2 trimer (green) in post-fusion state. (**c**) Superposition of the SARS-CoV S2 subunit, MHV S2 subunit[4] (PDB: 6B3O) and the predict SARS-CoV-2 S2 subunit in single chain mode. The cartoons are colored as in b.

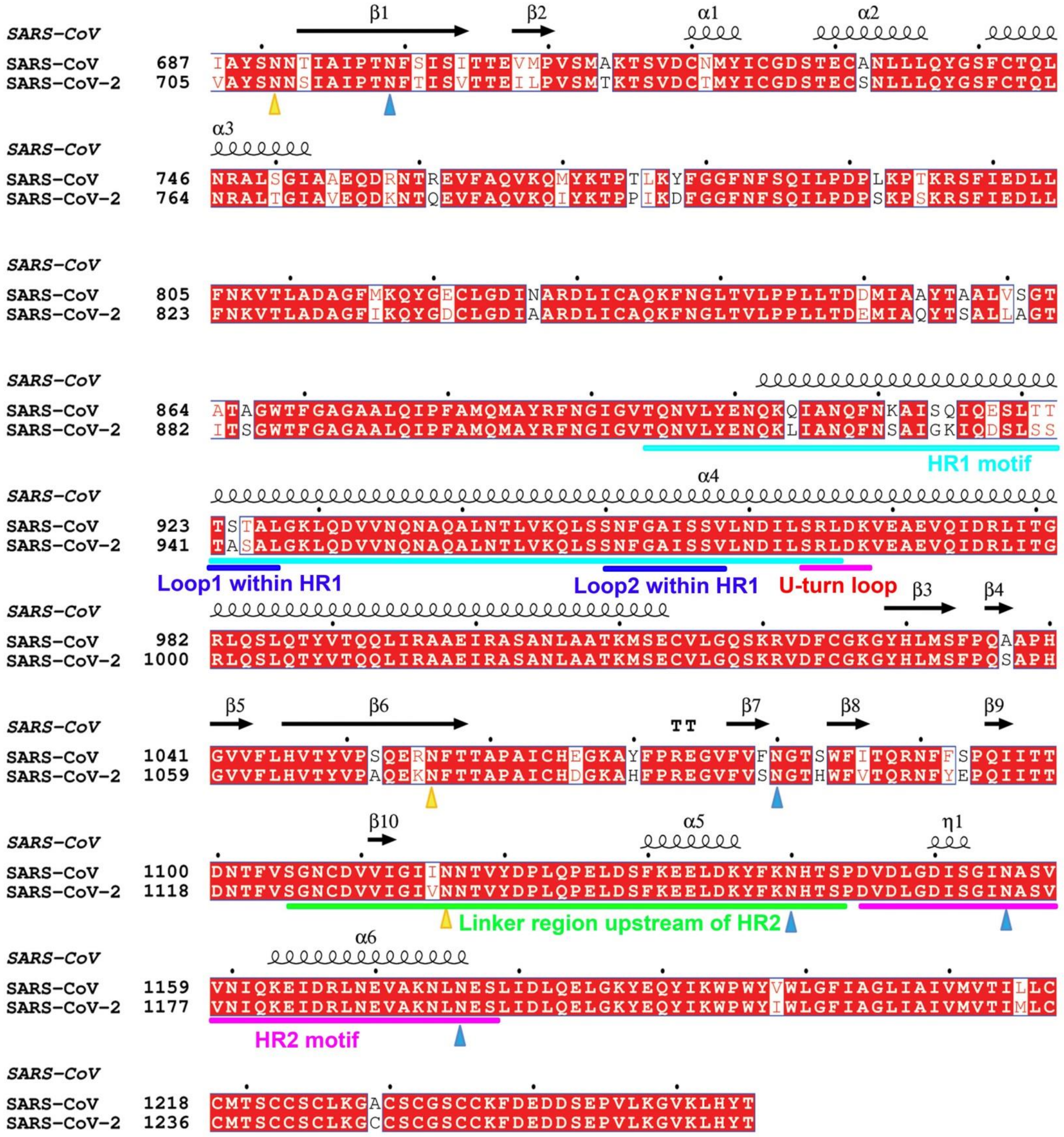

Supplementary Figure 6. Protein sequence conservation analysis of the S2 subunit from SARS-CoV and SARS-CoV-2. The potential therapeutic targets are labeled under the sequences. The glycosylation sites identified in our post-fusion SARS-CoV S2 structure are labeled with triangles (triangles represent glycosylation sites conserved among SARS-CoV, SARS-CoV-2, MERS-CoV and MHV are colored in blue, triangles represent glycosylation sites only conserved between SARS-CoV and SARS-CoV-2 are colored in yellow).

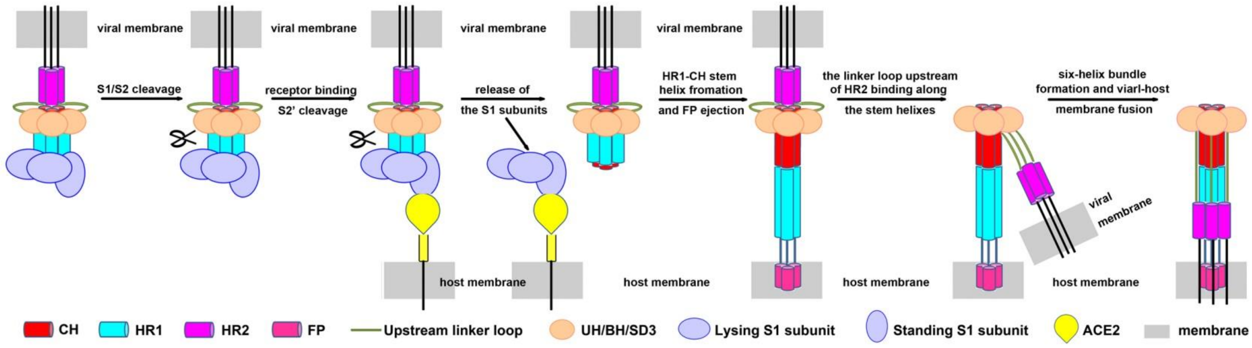

Supplementary Figure 7. Proposed mechanism of coronavirus membrane fusion process indicated by SARS-CoV S glycoprotein. Cleavage at the boundary of the S1 and S2 subunits dose not change the overall structure of the pre-fusion S trimer. Binding with host receptor and further cleavage at the S2' site accelerate the release of the S1 subunits. Conformational changes of the central helical region pumps the FP into the target membrane. The linker regions upstream of the HR2 motif are released and bind along the stem helices. The HR2 motifs are brought close to the HR1 motifs and form the six-helix bundle, which finally trigger membrane fusion.

Supplementary Table 1. Cryo-EM data collection, refinement and validation statistics

	**Post-fusion SARS-CoV S2 trimer (EMDB-30072) (PDB 6M3W)**
**Data collection and processing**	
Magnification	130,000
Voltage (kV)	200
Electron exposure (e–/Å^2)	50
Defocus range (μm)	1.8-2.2
Pixel size (Å)	1
Symmetry imposed	C3
Initial particle images (no.)	343,042
Final particle images (no.)	40,600
Map resolution (Å)	3.9
FSC threshold	0.143
Map resolution range (Å)	3.5-4.5
**Refinement**	
Initial model used (PDB code)	6B3O
Model resolution (Å)	4.0
FSC threshold	0.5
Model resolution range (Å)	3.5-4.5
Map sharpening *B* factor (Å^2)	-165
Model composition	
Non-hydrogen atoms	8307
Protein residues	1032
Ligands	24
B factors (Å^2)	
Protein	89
Ligand	126
R.m.s. deviations	
Bond lengths (Å)	0.01
Bond angles (°)	1.32
Validation	
MolProbity score	2.09
Clashscore	9.66
Poor rotamers (%)	0.66
Ramachandran plot	
Favored (%)	88.92
Allowed (%)	11.08
Disallowed (%)	0

Supplementary References:

1. Xu, Y. et al. Crystal structure of severe acute respiratory syndrome coronavirus spike protein fusion core. *J Biol Chem* **279**, 49414-9 (2004).
2. Xu, Y. et al. Structural basis for coronavirus-mediated membrane fusion. Crystal structure of mouse hepatitis virus spike protein fusion core. *J Biol Chem* **279**, 30514-22 (2004).
3. Gao, J. et al. Structure of the fusion core and inhibition of fusion by a heptad repeat peptide derived from the S protein of Middle East respiratory syndrome coronavirus. *J Virol* **87**, 13134-40 (2013).
4. Walls, A.C. et al. Tectonic conformational changes of a coronavirus spike glycoprotein promote membrane fusion. *Proc Natl Acad Sci U S A* **114**, 11157-11162 (2017).
